# The role of sheep ked (*Melophagus ovinus*) as potential vector of protozoa and bacterial pathogens

**DOI:** 10.1038/s41598-021-94895-x

**Published:** 2021-07-29

**Authors:** Joanna Werszko, Marek Asman, Joanna Witecka, Żaneta Steiner-Bogdaszewska, Tomasz Szewczyk, Grzegorz Kuryło, Konrad Wilamowski, Grzegorz Karbowiak

**Affiliations:** 1 Witold Stefański Institute of Parasitology, Polish Academy of Sciences, Twarda 51/55, 00-818 Warsaw, Poland; 2 Department of Parasitology, Faculty of Pharmaceutical Sciences in Sosnowiec, Medical University of Silesia, Jedności 8, 41-218 Sosnowiec, Poland; 3 Institute of Forest Sciences, Faculty of Civil Engineering and Environmental Sciences, Białystok University of Technology, Wiejska 45e, 15-351 Białystok, Poland

**Keywords:** Molecular biology, Parasitic infection, Entomology, Parasitology

## Abstract

The sheep ked (*Melophagus ovinus*) hematophagous insect may act as a potential vector of vector-borne pathogens. The aim of this study was to detect the presence of *Trypanosoma* spp., *Bartonella* spp., *Anaplasma phagocytophilum* and *Borrelia burgdorferi* sensu lato in sheep ked collected from sheep in Poland. In total, *Trypanosoma* spp. was detected in 58.91% of *M. ovinus,* whereas *Bartonella* spp. and *B. burgdorferi* s.l. were found in 86.82% and 1.55% of the studied insects, respectively. *A. phagocytophilum* was not detected in the studied material. In turn, co-infection by *Trypanosoma* spp. and *Bartonella* spp. was detected in 50.39%, while co-infection with *Trypanosoma* spp. and *Bartonella* spp. and *B. burgdorferi* s.l. was found in 1.55% of the studied insects. The conducted study showed for the first time the presence of *B. burgdorferi* s. l. in *M. ovinus,* as well as for the first time in Poland the presence of *Trypanosoma* spp. and *Bartonella* spp. The obtained results suggest that these insects may be a potential vector for these pathogens, but further-more detailed studies are required.

## Introduction

The family Hippoboscidae (Diptera) is a group of blood-sucking flies of veterinary importance that parasitize on mammals and birds. Worldwide, the fauna of Hippoboscinae consists of more than 213 species, 21 genera and three subfamilies: (Ornithomyinae, Hippoboscinae and Lipopteinae)^1,2^. In Europe, about 30 species of hippoboscid have been described, of which 10 species are found in Poland^2,3^. The sheep ked (*Melophagus ovinus*) is a blood-sucking wingless ectoparasite of sheep. Their life-cycle consists of three stages: larva, pupa and adult, and occurs in the fleece of the sheep host and can be carried from one sheep to another by direct contact^4^. Although the sheep ked parasitizes mainly sheep, their incidental occurrence has also been reported in red fox, rabbit and the European bison^4,5^. This species of flies occurs in North America, Oceania, Asia, China, Africa and Europe^4,6^. The sheep keds have an economic impact by reducing the production of meat, milk and the wool of sheep, and the infestation of sheep also has harmful effects, such as: weight loss, anemia, anxiety and reduction in wool growth. In addition, skin damage due to abrasion and scratching can lead to secondary microbiological infections^4^. The sheep ked has been reported to be responsible for the transmission of zoonotic pathogens, such as: *Bartonella* spp.*, Anaplasma* spp., bluetongue virus, border disease virus (BDV), *Rickettsia* spp., *Trypanosoma* spp.^6–13^. The occurrence of arthropod-borne pathogens in *M. ovinus* has not yet been studied in Poland.

Trypanosomes (genus *Trypanosoma,* family Trypanosomatidae, order Kinetoplastea), are flagellated protozoa with a worldwide distribution and have been isolated from the gut of sheep ked and the blood of sheep^14^. Due to the developmental mode, it belong to the Stercorarian group. The infection of trypanosomes in the mammalian host takes place through damaged skin or mucous membranes when the trypanosomes leave the insect organism with the faeces^14^. Trypanosomiasis in animals are usually subclinical, although anaemia, leucocytosis, weight loss, neonate death and decreased milk production have been noted^15^.

*Anaplasma phagocytophilum* causes granulocytic anaplasmosis in humans (HGE) as well tick-borne fever in ruminants, equine anaplasmosis in horses, and severe febrile diseases in dogs and cats^16^. It is Gram-negative intracellular bacteria of the family Anaplasmataceae that grow and multiply in the membrane-bound vacuoles of vertebrate and invertebrate host cells^17^. The disease is multi-systemic and causes lethargy, ataxia, loss of appetite, and weak or painful limbs. The cells most commonly infected are neutrophilic granulocytes. Humans can be the host, and the animal reservoir for *A. phagocytophilum* depends on the geographic region and includes many animal species, including wild animals such as rodents, carnivores, deer and domestic animals, such as cattle, goats, and sheep^18–20^. Molecular studies based on the 16S rRNA gene, two different genetic variants of *A. phagocytophilum* have been described. The AP-ha strain is pathogenic to both humans and animals, while the AP -variant 1 strain is infectious to animals but not to humans^21,22^. In Europe, zoonotic reservoirs of human *A. phagocytophilum* strains are wild boar, the hedgehog, and possibly carnivores^23,24^. The *Ixodes ricinus* tick is a primary vector of this bacteria, but their presence has been also confirmed in deer keds (*Lipoptena cervi*) nor in some species of blood-sucking flies from the Tabanidae family^25,26^.

*Bartonella* spp. are small, intracellular, hemotropic Gram-negative bacteria that cause long-lasting infections in their mammalian hosts and are mainly transmitted by arthropod vectors^27^. In the last 20 years, the number of *Bartonella* species descriped has increased rapidly, with at least 26 species now designated and with some species containing more than one subspecies^28^. The clinical manifestations of human bartonellosis depend on the species of the infecting *Bartonella,* and most often manifest as various cardiovascular, neurological and rheumatological conditions^28^. Tsai and co-authors^29^ showed that in recent decades a variety of insect vectors and mammal hosts have been associated with *Bartonella* sp. infections. *Bartonella* species were identified mainly based on PCR amplification in a wide range of hematophagous arthropods. These bacterial pathogens were detected in human lice (*Pediculus humanus*), cat fleas (*Ctenocephalides felis*), sand flies (*Lutzomyia verrucarum*), and various hard tick species, such as *Rhipicephalus sanguineus* and varoius species from genera *Ixodes* spp., *Dermacentor s*pp., *Haemaphysalis* spp.^30^.

Lyme disease, a mul-systemic, chronic, and often clinically diverse human disease is caused by the spirochetes of *Borrelia burgdorferi* sensu lato (Spirochaetia, order Spirochaetales, family Spirochaetaceae). The *B. burgdorferi* s.l. complex consist of 22 genospecies, of which 11 are circulating in Europe and five of them, namely *B. afzelii*, *B. garini*, *B. burgdorferii* sensu stricto., *B. spielmanii*, *B. bavariensis,* are pathogenic to humans and associated with human Lyme disease^31,32^. Blood-sucking flies, such as the deer fly, horse fly and flea, have been found to be infected with *B. burgdorferi* s.l. in Europe and North America^33,34^. These pathogens have been described recently in sheep ked in China^9^.

In the current study, a molecular screening was conducted of sheep keds for the presence of *Trypanosoma* spp. *Anaplasma* spp. *Bartonella* spp. and *Borrelia burgdorferi* s.l. pathogens in sheep ked collected from a sheep farm near the Białowieża Primeval Forest in north-eastern Poland. To the best of the authors’ knowledge, this is the first report to confirm the presence of these pathogens in sheep ked in Poland. Evidence is provided that two (*Trypanosoma* spp. and *Bartonella* spp.) and three pathogens (*Trypanosoma* spp. and *Bartonella* spp. and *B. burgdorferi* s.l.) can co-infect sheep ked (*Melophagus ovinus*).

## Results

**Figure 1:**
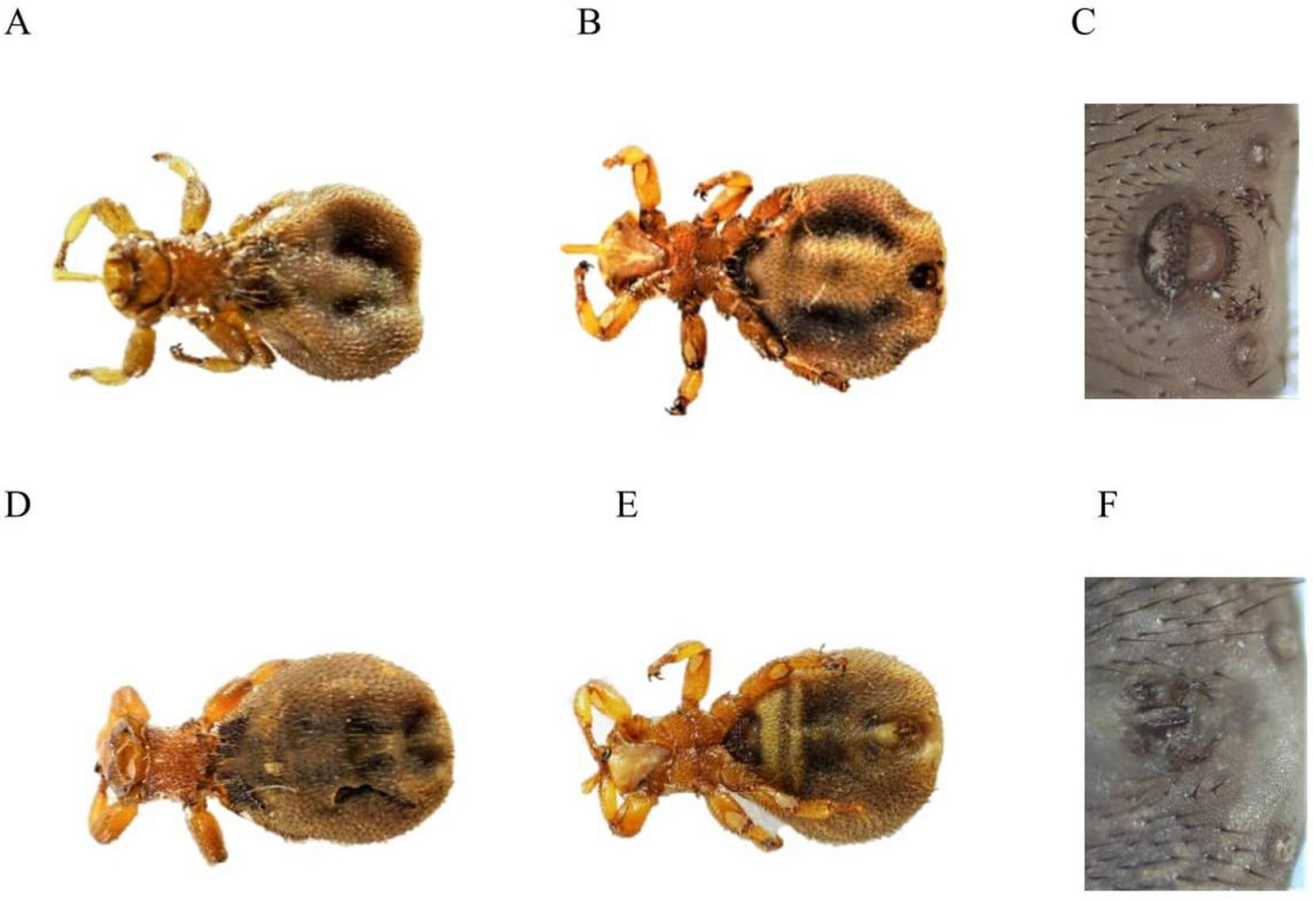
Female of *Melophagus ovinus* dorsal (**A**) and ventral view (**B**), posterior end (**C**) and male of *Melophagus ovinius* dorsal (**D**) and ventral view (**E**); Posterior end (**F**).

**Figure 2:**
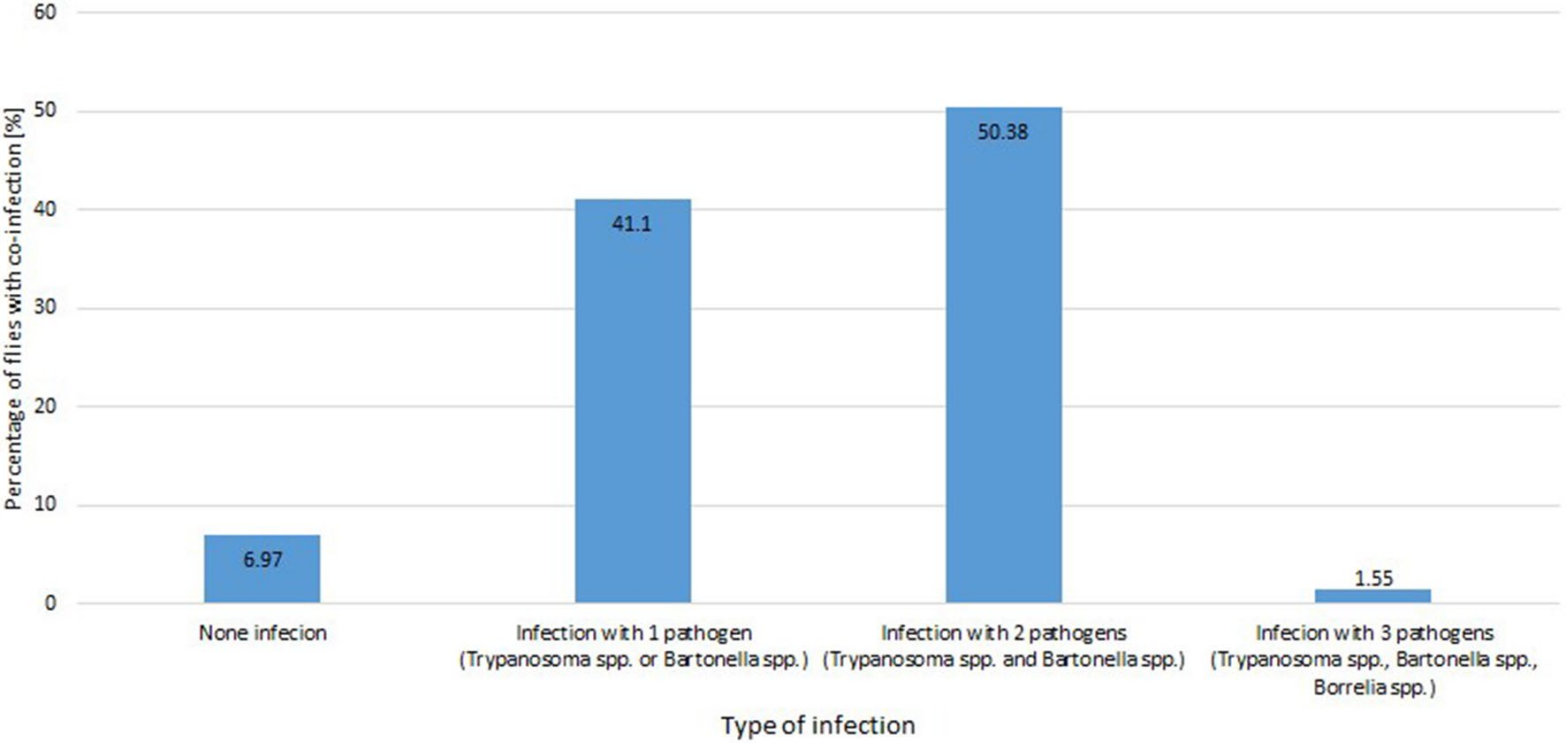
Total percentage of co-infection with *Trypanosoma* spp., *Bartonella s*pp. and *Borrelia burgdorferi* s.l. in flies collected from the studied areas.

In total, 129 *M. ovinus* (69 ♀; 60 ♂) were collected from sheep (Fig. 1)The presence *Trypanosoma* spp. was detected in 76 of the 129 (58.91%) studied insects. In turn, *Bartonella* spp. was found in 122 of the 129 (86.82%) flies. The presence of *B. burgdorferi* s.l*.* was detected in 2 of the 129 (1.55%) sheep keds, whereas no *A. phagocytophilum* was detected in the tested group of insects. The positivity to protozoan and bacterial pathogens DNA among female and male *M. ovinus* from different locations is given in Table 1. Co-infection with the two pathogens *Trypanosoma* spp. and *Bartonella* spp was detected in 65 of the 129 (50.39%) *M. ovinus*, whereas co-infection with *Trypanosoma* spp. and *Bartonella* spp. and *B. burgdorferi* s.l was detected in only 2 of the 129 (1.55%) sheep keds (Fig. 2). In the current study, *A. phagocytophilum* was not detected in the tested group of flies.

**Table 1:** Specimens of *Melophagus ovinus* analysed for *Trypanosoma* spp., *Bartonella* spp., *Borrelia burgdorferi* s.l. and *Anaplasma phagocytophilum* infection.

The prevalence of *pathogens* infections in %			Location				Overall
			Rzepiska	Nowoberezowo	Waliły	Gródek	
*Trypanosoma* spp.	*M. ovinus* ♀	Analyzed	23	28	18	0	69
		Infected	21	7	14	0	42
	*M. ovinus* ♂	Analyzed	15	11	15	19	60
		Infected	15	2	0	17	34
	Total	Analyzed	38	39	33	19	129
		Infected	36	9	14	17	76 (58.91%)
*Bartonella* spp*.*	*M. ovinus* ♀	Analyzed	23	28	18	0	69
		Infected	23	24	18	0	65
	*M. ovinus* ♂	Analyzed	15	11	15	19	60
		Infected	12	8	13	14	47
	Total	Analyzed	38	39	33	19	129
		Infected	35	32	31	14	112 (86.82%)
*Borrelia burgdorferi* s.l	*M. ovinus*♀	Analyzed	23	28	18	0	69
		Infected	0	0	0	0	0
	*M. ovinus* ♂	Analyzed	15	11	15	19	60
		Infected	0	0	0	2	2
	Total	Analyzed	38	39	33	19	129
		Infected	0	0	0	2	2 (1.55%)
*Anaplasma* spp*.*	*M. ovinus*♀	Analyzed	23	28	18	0	69
		Infected	0	0	0	0	0
	*M. ovinus* ♂	Analyzed	15	11	15	19	60
		Infected	0	0	0	0	0
	Total	Analyzed	38	39	33	19	129
		Infected	0	0	0	0	0

Sequence analysis showed that four partial *rpoB* gene sequences of *Bartonella* spp. (Gen Bank Accession No., MW929188, MW929189, MW929190, MW92919) were identical to each other and showed 100% identity with *Bartonella melophagi* from sheep ked from the USA (EF605288). In total, eight partial 18S rDNA sequences of *Trypanosom*a spp. were obtained in the current study, three of which (Acc. No., MZ014571, MZ014572, MZ014565) shared 100% similarity with *Trypanosoma melophagium* from *M. ovinus* in the United Kingdom and Croatia (FN666409, HQ664912). While the other five sequences (Acc. No., MZ014566, MZ014567, MZ014568, MZ014569, MZ014570) showed 100% identity with *Trypanosoma* sp. from *Lipoptena fortisetosa* in Poland (MT393991). Analysis of two partial *fla B* gene nucleotide sequences of *B. burgdorferi* s.l. (GenBank Acc. No., MW929186, MW929187) obtained in this study showed that they were identical to each other, and shared 100% similarity with *B. burgdorferi* strains from *Ixodes ricinus* ticks in Poland (MK604273, MK604272, MF150052, KX64620, KF422802, HM345911), Switzerland (KF422803) and Serbia (AB189460) and with *B. burgdorferi* isolates obtained from the human serum of a patient with Lyme in the Czech Republic (FJ231333–FJ231335). This result confirm the presence of strain typical for Middle Europe. Moreover, they were also identical with *B. burgdorferi* from cotton mouse (*Peromyscus gossypinus*) from the USA (EU220782).

Statistical comparisons (Table 2) revealed that there were three significant associations between variables, i.e., for prevalence of *Trypanosoma* spp. on *M. ovinus* and sampling location (*X*^2^ = 51.8768, p ≤ 0.01) and for prevalence of any of examined pathogens on *M. ovinus* and sampling location (*X*^2^ = 15.6845, p ≤ 0.01) as well as for prevalence of *Bartonella* spp. on *M. ovinus* and subject gender (*X*^2^ = 7.064, p ≤ 0.01). All of the other associations tested were not statistically significant.

**Table 2:** Statistics of the chi-square tests performed to examine associations between prevalences of (i) *Trypanosoma* spp., (ii) *Bartonella* spp., (iii) *Trypanosoma* spp., *Bartonella* spp., *Borrelia burgdorferi* s.l. or *Anaplasma phagocytophilum* on *Melophagus ovinus* and (i) sampling location, (ii) sex of *M. ovinus*.

Variables	*Melophagus ovinus* infected/non-infected by
Sampling location (I—Waliły, II—Gródek, III—Rzepiska, IV—Nowoberezowo)	*Trypanosoma* spp. *X*^2^ (3, *N* = 129) = 51.8768*, *p* < 0.00001
	*Bartonella* spp. *X*^2^ (3, *N* = 129) = 6.0301, *p* = 0.11015
	*Trypanosoma* spp., *Bartonella* spp., *Borrelia burgdorferi* s.l. or *Anaplasma phagocytophilum* *X*^2^ (3, *N* = 516) = 15.6845*, *p* = 0.00132
Sex of *M. ovinus* (♀—females, ♂—males)	*Trypanosoma* spp. *X*^2^ (1, *N* = 129) = 0.2342, *p* = 0.62842
	*Bartonella* spp. *X*^2^ (1, *N* = 129) = 7.064*, *p* = 0.00786
	*Trypanosoma* spp., *Bartonella* spp., *Borrelia burgdorferi* s.l. or *Anaplasma phagocytophilum* *X*^2^ (1, *N* = 516) = 0.9664, *p* = 0.32559

*The result is significant at *p* ≤ 0.01.

## Discussion

The sheep keds in this study were collected in north-eastern Poland, recognized as an endemic region with a high risk of tick-borne disease infection. Despite that, in Poland there is no official data about sheep ked and their role in the transmission of pathogens and their vector competency for infectious agents. The study records for the first time in Poland the presence of a protozoan (*Trypanosoma* spp.) and bacteria (*Bartonella* spp., *B. burgdorferi* s.l. and *A. phagocytophilum*) in sheep ked by using molecular methods. The results obtained indicate a high positivity of *Trypanosoma* spp. infection among sheep ked collected in Poland (58.91%), although a higher prevalence (82.4%) of trypanosomes was recorded for St Kilda sheep ked in the Outer Hebrides, Scotland^35^.

In the current study, a high frequency of *Bartonella* spp. (86.82%) in *M. ovinus* was found, with similar results obtained by Halos et al.^30^ who detected this bacteria in all specimens in the tested group of sheep ked in Europe. Similarly, Kumsa et al.^11^ noted a high positivity of *B. melophagi* infection (88.6%) in sheep ked collected in Africa. Moreover, Rudolf et al.^12^ identified *B. melophagi* in sheep keds in Central Europe, in which all investigated pools (399 specimens) of keds were positive for *Bartonella* spp. Similarly, in Algeria, 36.87% of sheep ked were infected with *Bartonella* spp.^13^.

The results obtained in the current study show for the first time in Europe evidence of *B. burgdorferi* s.l. in *M. ovinus* collected from sheep. However, this bacteria was evident in only 1.5% of the studied insects. The study conducted in China by Chu et al.^9^ showed a twice as high percentage in *M. ovinus* infected with *B. garinii* and *B. valaisiana* (related group of *B. burgdorferi* s.l).

In the presented study, DNA of *A. phagocytophilum* was not detected in the tested group of flies, while Hornok et al.^8^ detected the presence DNA of *Anaplasma ovis* in all (81 specimens) of sheep ked collected from sheep in Hungary. In Algieria, 25.88% of the *M. ovinus* were infected by bacteria from the Anaplasmataceae family^13^. In a study conducted by Zhang et al.^36^ the infection rates were 39.1%, 17.4%, and 9.8% for *A. ovis*, *A. bovis*, and *A. phagocytophilum* in *M. ovinus*, respectively.

The mixed infection of sheep keds with two bacterial genus, *Bartonella* and *Anaplasma,* were noted, with frequency co-infection at 18.64%^13^. The obtained results also demonstrated that two pathogens, *Trypanosoma* spp. and *Bartonella* spp., can co-infect the same sheep ked.

The presence of *Trypanosoma* spp., *Bartonella* spp., and *B. burgdorferi* s.l. pathogens in *M. ovinus* collected from sheep may be related to their biology. The sheep ked spends its entire life cycle on the host; however, adults may circulate among the animals of the same herd, and are commonly transferred from ewes to their offspring^4^. Hippoboscids are also likely to be mechanical vectors of infectious agents due to their blood feeding behavior^37^. Some pathogens may be transferred from an infected host to non-infected individuals through their mouthparts^38^, although the keds were probably infected through receiving the pathogens from sheep. However, it is interesting that keds and sheep are generally thought to be reservoirs incompetent for *B. burgdorferi* s.l.^9,39^. In the current study, the keds probably acquired the *B. burgdorferi* s.l infections via co-feeding transmission when one infected *Ixodes* spp. tick infected a ked feeding nearby. At this stage of the study, it is difficult to explain this speculation. This is the first report in Europe on the detection of *B. burgdorferi* s.l. DNA in sheep keds.

Recently, vertical transmission of *A. ovis* in sheep keds has been described^13,40^. The presence of *Bartonella* DNA in all of the *M. ovinus* samples, even at the pupal stage, has been described^30^. This provides new evidence for the potential of these flies as mechanical or biological vectors^13^.

Due to the results of statistically significant comparisons (chi-square tests) between prevalence of *Trypanosoma* spp. and *Bartonella* spp. on *M. ovinus* versus sampling location and sex of the flie further research on this subject should be carried out.

In conclusion, this study provides the first molecular evidence of the presence of DNA of *Trypanosoma* spp, *Bartonella* spp., *B. burgdoreferi* s.l. in a tested group of sheep ked. Detection of the pathogen in *M. ovinus* is important evidence that other blood-sucking flies, including hippoboscid flies, are important candidates as potential vectors of infectious diseases.

## Materials and methods

Adult *M. ovinus* were collected during veterinary procedures from sheep on four sheep farms located near forest areas in north-eastern Poland, located in the villages of Rzepiska (52° 49′ 56″ N, 23° 33′ 56″ E), Nowoberezowo (52° 45′ 36″ N, 23° 33′ 43″ E), Waliły (53° 08′ 30″ N, 23° 35′ 25″ E), Gródek (52° 05′ 48″ N, 23° 39′ 24″ E). After collection, the flies were preserved in pure 70% ethanol for further morphological and molecular processing. Before identification, specimens were rinsed in pure water (Direct-Pure^®^ adept Ultrapure Lab Water Systems, RephiLe Bioscience, Ltd. China) and air-dried. Gender determination and species identification were carried out using taxonomic keys, according to Borowiec^3^ under an OPTA-TECH microscope (Warsaw, Poland).

The DNA was isolated from single individuals by using a Genomic Mini AX Tissue kit (A&A Biotechnology, Gdynia, Poland), according to the manufacturer’s recommended protocol. Concentration was measured spectrophotometrically at 260/280 nm wave length. Isolated material was stored at − 20 °C until further molecular analysis. Pathogens in *M. ovinus* were detected by PCR and nested PCR methos.

The detection of *Trypanosoma* spp. was based on PCR amplification of a 18S rDNA gene fragment of approximately 650 bp. Two oligonucleotide primers, TrypF 150 and TrypR 800, were used, as previously described^41^. For amplification, a 200 ng DNA template was used, and for the reactions Allegro *Taq* DNA Polymerase (Novazym, Poznań, Poland), was used, and to detect *Bartonella* spp., a pair of primers—1400F and 2300R—were used to amplify an 850 bp fragment of the *rpoB* gene^42^. PCR reactions were conducted according to Paziewska et al.^43^. For the reaction mixture, RUN *Taq* polymerase (A&A Biotechnology, Gdynia, Poland) was used.

In turn, *B. burgdorferi* s.l., was detected in insects with the use of the two pairs of primers specific to the *fla B* gene fragment, as previously described^44^. For amplification, a 200 ng DNA template was used. In turn, for the re-amplification, 1 µl of the amplification product was used. DFS-Plus DNA Taq Polymerase (GeneOn, Germany) was used for both reactions. The presence of 824 bp and 605 bp reaction products were considered positive.

*Anaplasma phagocytophilum* was detected in *M. ovinus* with the use of two pairs of primers specific to the 16S rDNA gene fragment, as previously described^45^. A 200 ng DNA template and 1 µl of amplification product were used for amplification and re-amplification, respectively. For both reactions, Taq DNA Polymerase (EURx, Poland) was used. The presence of 932 bp and 546 bp reaction products were considered positive.

PCR and nested PCR products were visualized on 1% and 2% ethidium bromide stained agarose gels. Next, gels were visualized using ChemiDoc, MP Lab software (Imagine, BioRad, Hercules, USA) or Omega 10 (UltraLum, USA) and TotalLab software (TotalLab, UK). The positive products of PCR and nested PCR were purified using the QIAEX II Gel extraction kit (Qiagen, Hilden, Germany) or Agarose-Out DNA Purification Kit (EURx, Poland), and sequenced by Genomed (Warsaw, Poland). Next, the sequences were assembled into contigs using ContigExpress, Vector NTI Advance 11.0 (Invitrogen Life Technologies, New York, USA). The sequences were then aligned with reference sequences available in GenBank by BLAST (BasicLocal Alignment Search Tool) and analyzed using MEGA 5.0 software.

*X*^2^ statistical analyses were performed to examine the relation between the prevalence data and: (i) sampling locations as well as (ii) sex of *M. ovinus*, and the 5% level of probability was set for rejection of the null hypothesis.

### Ethics approval and consent to participate

Not applicable.

## References

[1] Dick, C. W. *Checklist of World Hippoboscidae (Diptera: Hippoboscoidea)*. 1–7 (Department of Zoology, Field Museum of Natural History, 2006).

[2] Petersen, F. T. Fauna Europaea: Hippoboscidae. In *Fauna Europaea: Diptera, Brachycera.* (eds. Beuk, P. & Pape, T.) (Fauna Europaea, 2013). 2.6. https://fauna-eu.org. Accessed 10 July 2019.

[3] Borowiec, L. *Wpleszczowate—Hippoboscidae. Klucze do oznaczania owadów Polski Cz. 28, z. 21*. (Wydawnictwo PWN, 1984).

[4] Small RW (2005). A review of *Melophagus ovinus* (L.), the sheep ked. Vet. Parasitol..

[5] Karbowiak G, Demiaszkiewicz AW, Pyziel AM, Wita I, Moskwa B, Werszko J, Bień J, Goździk K, Lachowicz J, Cabaj W (2014). The parasitic fauna of the European bison (*Bison bonasus*) (Linnaeus, 1758) and their impact on the conservation. Part 1. The summarising list of parasites noted. Acta Parasitol..

[6] Liu D, Wang YZ, Zhang H, Liu ZQ, Wureli HZ, Wang SW, Tu CC, Chen CF (2016). First report of *Rickettsia raoultii* and *R. slovaca* in *Melophagus ovinus*, the sheep ked. Parasit. Vectors..

[7] Luedke AJ, Jochim MM, Bowne JG (1965). Preliminary blue-tongue transmission with the sheep ked *Melophagus ovinus* (L.). Can Jo Com Med. Vet. Sci..

[8] Hornok S, de la Fuente J, Biro N, de Mera IGF, Meli LM, Elek V, Gonczi E, Meili T, Tanczos B, Farkas R, Lutz H, Hofmann-Lehmann R (2011). First molecular evidence of *Anaplasma ovis* and *Rickettsia* spp. in keds (Diptera: Hippoboscidae) of sheep and wild ruminants. Vector Borne Zoonotic Dis..

[9] Chu CY, Jiang BG, Qiu EC, Zhang F, Zuo SQ, Yang H, Liu W, Cao WC (2011). *Borrelia burgdorferi* sensu lato in sheep keds (*Melophagus ovinus*), Tibet, China. Vet. Microbiol..

[10] Martinković F, Matanović K, Rodrigues AC, Garcia HA, Teixeira MMG (2012). *Trypanosoma* (*Megatrypanum*) *melophagium* in the Sheep Ked *Melophagus ovinus* from Organic Farms in Croatia: Phylogenetic inferences support restriction to sheep and sheep keds and close relationship with trypanosomes from other ruminant species. J. Eukaryot. Microbiol..

[11] Kumsa B, Parola P, Raoult D, Socolovschi C (2014). *Bartonella melophagi* in *Melophagus ovinus* (sheep ked)collected from sheep in northern Oromia, Ethiopia. Comp. Immunol. Microbiol. Infect. Dis..

[12] Rudolf I, Betasova L, Bischof V, Venclikova K, Blazejova H, Mendel J, Hubalek Z, Kosoy M (2016). Molecular survey of arthropod-borne pathogens in sheep keds (*Melophagus ovinus*), Central Europe. Parasitol. Res..

[13] Boucheikhchoukh M, Mechouk N, Benakhla A, Raoult D, Parola P (2019). Molecular evidence of bacteria in Melophagus ovinus sheep keds and *Hippobosca equina* forest flies collected from sheep and horses in northeastern Algeria. Comp. Immunol. Microbiol. Infect. Dis..

[14] Hoare CA (1972). The Trypanosomes of Mammals.

[15] Matsumoto Y, Sato A, Hozumi M, Ohnishi H, Kabeya M, Sugawara M, Takaishi H (2011). A case of a Japanese Black cow developing trypanosomosis together with enzootic bovine leucosis. J. Jpn. Vet. Med. Assoc..

[16] Stuen S, Granquist EG, Silaghi C (2013). *Anaplasma phagocytophilum*-a widespread multi-host pathogen with highly adaptive strategies. Front. Cell Infect. Microbiol..

[17] Dumler JS, Barbet AF, Bekker CP, Dasch GA, Palmer GH, Ray SC, Rikihisa Y, Rurangirwa FR (2001). Reorganization of genera in the families Rickettsiaceae and Anaplasmataceae in the order Rickettsiales: Unifiation of some species of Ehrlichia with Anaplasma, Cowdria with Ehrlichia and Ehrlichia with Neorickettsia, descriptions of six new species combinations and designation of *Ehrlichia equi* and ‘HGE agent’ as subjective synonyms of *Ehrlichia phagocytophila*. Int. J. Syst. Evol. Microbiol..

[18] Bakken JS, Krueth J, Tilden RL, Dumler JS, Kristiansen BE (1996). Serological evidence of human granulocytic ehrlichiosis in Norway. Eur. J. Clin. Microbiol. Infect. Dis..

[19] Dumler JS, Choi KS, Garcia-Garci AJC, Barat NS, Scorpio DG, Garyu JW, Grab DJ, Bakken JS (2005). Human granulocytic anaplasmosis and *Anaplasma phagocytophilum*. Emerg. Infect. Dis..

[20] Nicholson WL, Allen KE, McQuiston JH, Breitschwerdt EB, Little SE (2010). The increasing recognition of rickettsial pathogens in dogs and people. Trends Parasitol..

[21] Levin ML, Nicholson WL, Massung RF, Sumner JW, Fish D (2002). Comparison of the reservoir competence of medium-sized mammals and *Peromyscus leucopus* for *Anaplasma phagocytophilum* in Connecticut. Vector Borne Zoonotic Dis..

[22] Dugat T, Chastagner A, Lagrée AC, Petit E, Durand B, Thierry S, Corbière F, Verheyden H, Chabanne L, Bailly X, Leblond A, Vourc'h G, Boulouis HJ, Maillard R, Haddad N (2014). A new multiple-locus variable-number tandem repeat analysis reveals different clusters for *Anaplasma phagocytophilum* circulating in domestic and wild ruminants. Parasit. Vectors..

[23] Michalik J, Stańczak J, Cieniuch S, Racewicz M, Sikora B, Dabert M (2012). Wild boars as hosts of human-pathogenic *Anaplasma phagocytophilum* variants. Emerg. Infect. Dis..

[24] Karbowiak G, Biernat B, Stańczak J, Werszko J, Wróblewski P, Szewczyk T, Sytykiewicz H (2016). The role of particular ticks developmental stages in the circulation of tick-borne pathogens in Central Europe. 4. Anaplasmataceae. Ann. Parasitol..

[25] Víchová B, Majláthová V, Nováková M, Majláth I, Čurlík J, Bona M, Peťko B (2011). PCR detection of re-emerging tick-borne pathogen, *Anaplasma phagocytophilum*, in deer ked (*Lipoptena cervi*) a blood-sucking ectoparasite of cervids. Biologia.

[26] Werszko J, Szewczyk T, Steiner-Bogdaszewska Ż, Laskowski Z, Karbowiak G (2019). Molecular detection of *Anaplasma phagocytophilum* in blood-sucking flies (Diptera: Tabanidae) in Poland. J. Med. Entomol..

[27] Chomel BB, Boulouis HJ, Breitschwerdt EB, Kasten RW, Vayssier-Taussat M, Birtles RJ, Koehler JE, Dehio C (2009). Ecological fitness and strategies of adaptation of Bartonella species to their hosts and vectors. Vet. Res..

[28] Maggi RG, Mozayeni BR, Pultorak EL, Hegarty BC, Bradley JM, Correa M, Breitschwerdt EB (2012). *Bartonella* spp. bacteremia and rheumatic symptoms in patient from Lyme disease-endemic region. Emerg. Infect. Dis..

[29] Tsai YL, Chang CC, Chuang ST, Chomel BB (2011). *Bartonella* species and their ectoparasites: Selective host adaptation or strain selection between the vector and the mammalian host?. Comp. Immunol. Microbiol. Infect. Dis..

[30] Halos L, Jamal T, Maillard R, Girard B, Guillot J, Chomel B, Vayssier-Taussat M, Boulouis H-J (2004). Role of Hippoboscidae flies as potential vector of *Bartonella* spp. infecting wild domestic ruminants. Appl. Environ. Microbiol..

[31] Karbowiak G, Biernat B, Stańczak J, Werszko J, Szewczyk T, Sytykiewicz H (2018). The role of particular ticks developmental stages in the circulation of tick-borne pathogens in Central Europe. 5. Borreliaceae. Ann. Parasitol..

[32] Răileanu C, Tauchmann O, Vasić A, Wöhnke E, Silagh C (2020). *Borrelia miyamotoi* and *Borrelia burgdorferi* (*sensu lato*) identifcation and survey of tick-borne encephalitis virus in ticks from north-eastern Germany. Parasit. Vectors..

[33] Magnarelli LA, Anderson JF, Barbou RAG (1986). The etiologic agent of Lyme disease in deer flies, horse flies, and mosquitoes. J. Infect. Dis..

[34] Magnarelli LA, Anderson JF (1988). Ticks and biting insects infected with the etiologic agent of Lyme disease, *Borrelia burgdorferi*. J. Clin. Microbiol..

[35] Gibson W, Pilkington JG, Pemberton JM (2010). *Trypanosoma melophagium* from the sheep ked *Melophagus ovinus* on the island of St Kilda. Parasitology.

[36] Zhang Q-X, Wang Y, Li Y, Han S-Y, Wang B, Yuan G-H, Zhang P-Y, Yang Z-W, Wang S-L, Chen J-Y, Zhong H-S, Han X-Q, He H-X (2021). Vector-borne pathogens with veterinary and public health significance in *Melophagus ovinus* (Sheep Ked) from the Qinghai-Tibet Plateau. Pathogens..

[37] Bezerra-Santos MA, Otranto D (2020). Keds, the enigmatic flies and their role as vectors of pathogens. Acta Trop..

[38] Foil LD, Gorham R, Eldridge BF, Edman JD (2000). Mechanical transmission of disease agents by arthropods. Journal of Medical Entomology.

[39] Kurtenbach K, Sewell HS, Ogden NH, Randolph SE, Nuttall PA (1998). Serum complement sensitivity as a key factor in Lyme disease ecology. Infect. Immun..

[40] Zhao L, He B, Li KR, Li F, Zhang LY, Li XQ, Liu YH (2018). First report of *Anaplasma ovis* in pupal and adult *Melophagus ovinus* (sheep ked) collected in South Xinjiang, China. Parasit. Vector..

[41] Werszko J, Szewczyk T, Steiner-Bogdaszewska Ż, Wróblewski P, Karbowiak G, Laskowski Z (2020). Molecular detection of *Megatrypanum* trypanosomes in tabanid flies. Med. Vet. Entomol..

[42] Renesto P, Gouvernet J, Drancourt M, Roux V, Raoult D (2001). Use of *rpoB* analysis for detection and identification of *Bartonella* species. J. Clin. Microbiol..

[43] Paziewska A, Harris PD, Zwolińska L, Bajer A, Siński E (2011). Recombination within and between species of the alpha proteobacterium *Bartonella* infecting rodents. Microb. Ecol..

[44] Wodecka B, Rymaszewska A, Sawczuk M, Skotarczak B (2009). Detectability of tick-borne agents DNA in the blood of dogs undergoing treatment for borreliosis. Ann. Agric. Environ. Med..

[45] Massung RF, Slater KG (2003). Comparison of PCR assays for detection of the agent of human granulocytic ehrlichiosis, *Anaplasma phagocytophilum*. J. Clin. Microbiol..

